# NOD1 and NOD2 stimulation triggers innate immune responses of human periodontal ligament cells

**DOI:** 10.3892/ijmm.2012.878

**Published:** 2012-01-03

**Authors:** DO-IN JEON, SE-RA PARK, MEE-YOUNG AHN, SANG-GUN AHN, JONG-HWAN PARK, JUNG-HOON YOON

**Affiliations:** 1 Department of Pathology and Research Center for Oral Disease Regulation of the Aged, School of Dentistry, Chosun University, Gwangju 501-759; 2 Department of Biochemistry, College of Medicine, Konyang University, Daejeon 302-718, Republic of Korea

**Keywords:** nucleotide-binding oligomerization domain 1, nucleotide-binding oligomerization domain 2, immune response, periodontal ligament cells

## Abstract

Nod-like receptors (NLRs) are cytosolic sensors for microbial molecules. Nucleotide-binding oligomerization domain (NOD)1 and NOD2 recognize the peptidoglycan derivatives, *meso*-diaminopimelic acid (*meso*-DAP) and muramyl dipeptide (MDP), respectively, and trigger host innate immune responses. In the present study, we examined the function of NOD1 and NOD2 on innate immune responses in human periodontal ligament (PDL) cells. The gene expression of NOD1 and NOD2 was examined by RT-PCR. IL-6 and IL-8 production in culture supernatants was measured by ELISA. Western blot analysis was performed to determine the activation of NF-κB and MAPK in response to Tri-DAP and MDP. The genes of NOD1 and NOD2 appeared to be expressed in PDL cells. Although the levels of NOD2 expression were weak in intact cells, MDP stimulation increased the gene expression of NOD2 in PDL cells. Tri-DAP and MDP led to the production of IL-6 and IL-8 and the activation of NF-κB and MAPK in PDL cells. Toll-like receptor (TLR) stimulation led to increased gene expression of NOD1 and NOD2 in PDL cells. Pam_3_CSK_4_ (a TLR2 agonist) and IFN-γ synergized with Tri-DAP and MDP to produce IL-8 and IL-6 in PDL cells. Our results indicate that NOD1 and NOD2 are functionally expressed in human PDL cells and can trigger innate immune responses.

## Introduction

Host defense is achieved by two different immune systems, innate and adaptive immunity. Innate immunity is the first line of defense process to protect the host from microbial pathogens and is primarily mediated by phagocytes such as macrophages and dendritic cells (1–4). The innate immunity recognizes microorganisms via a limited number of pattern-recognition receptors (PRRs), which recognize microbial components, known as pathogen-associated molecular patterns (PAMPs). Toll-like receptors (TLRs) and Nod-like receptors (NLRs) are the representative PRRs.

They recognize microbial molecules including bacterial lipoprotein, lipopolysaccharide (LPS), flagellin, and viral nucleic acids, at the cell surface or endosomal membrane and subsequently activate NF-κB and MAPK to trigger the inflammatory process (1). In addition to microbial molecules, a variety of endogenous ligands, such as heat shock proteins, high mobility group box 1 (HMGB1), hyaluronan fragments, heparin sulphate, and fibronectin are recognized by TLR2 or TLR4 (5). In contrast, NLRs are intracellular, cytoplasmic sensors for microbial components and danger signals (6,7). There are 23 NLR family members in humans and at least 34 NLR genes in mice (7). NLRs are expressed in non-immune cells including epithelial and mesothelial cells as well as immune cells. Nucleotide-binding oligomerization domain (NOD)1 and NOD2, the first identified NLRs consist of an N-terminal caspase recruitment domain (CARD), an intermediate NOD, and a C-terminal leucine-rich repeats (LRRs) domain (8). NOD1 and NOD2 recognize the peptidoglycan derivatives, *meso*-diaminopimelic acid (*meso*-DAP) and muramyl dipeptide (MDP), respectively (8). After stimulation by their specific bacterial molecules, NOD1 and NOD2 associate with the adaptor molecule, RICK/Rip2/CARDIAK, through CARD-CARD interaction, which leads to activation of NF-κB and MAPK, followed by induction of numerous genes involved in the inflammatory process (9–11).

Periodontitis is a chronic inflammatory disease initiated on the periodontium by toxin and oxygen produced from periodontopathic bacteria (12), which results in tooth loss and periodontal bone resorption because the supportive tissue surrounding the teeth was destructed. Gram-negative bacteria such as *Porphyromonas gingivalis*, *Aggregatibacter actinomycetemcomitans* and *Fusobacterium necleatum* have been considered to be important pathogenic microorganisms associated with periodontitis (12–14).

Periodontal ligament (PDL) cells not only function as supporting cells for periodontal tissues but also produce inflammatory mediators that recognize various molecules including LPS (15). There is evidence that TLRs mediate immune responses of PDL cells against periodontal infections (16,17). However, little is known about the role of NOD1 and NOD2 in innate immune responses of PDL cells. Therefore, in the present study, we examined the function of NOD1 and NOD2 in the innate immune responses of human PDL cells.

## Materials and methods

### Cell culture and reagents

A human periodontal ligament cell line was a gift from Dr Maeda (Kyushu University Hospital, Fukuoka, Japan). This cell line was immortalized by SV40 T-antigen and hTERT gene transfer (18). THP-1 cells, a human monocytic leukemia cell line, were used as a positive control. PDL cells were cultured in Minimum Essential Medium α (Gibco, Grand Island, NY, USA) containing 1X penicillin/streptomycin and 10% fetal bovine serum in 5% CO_2_ incubator at 37°C. Tri-DAP, Pam_3_CSK_4_, LPS, and recombinant human IFN-γ were purchased from Invivogen, Inc. (San Diego, CA, USA) and muramyl dipeptide [MDP; Ac-(6-O-strearoyl)-muramyl-Ala-D-Glu-NH_2_] was from Bachem, Inc. (Torrance, CA, USA).

### RT-PCR

Total-RNA was extracted from the cell using easy-BLUE (Intron Biotechnology, Daejeon, Korea) according to the manufacturer’s instruction. One microgram of total-RNA was reverse transcribed into cDNA, and PCR was performed using the Power cDNA Synthesis kit (Intron Biotechnology) and One-step RT-PCR with AccuPower^®^ HotStart PCR PreMix (Bioneer, Daejeon, Korea). The following primer sets were used. Human NOD1, forward, 5′-CCACTTCACAGCTGGAG ACA-3′ and reverse, 3′-TGAGTGGAAGCAGCATTTTG-5′; human NOD2, forward, 5′-GAATGTTGGGCACCTCAAGT-3′ and reverse, 3′-CAAGGAGCTTAGCCATGGAG-5′; human GAPDH, forward, 5′-GTCGGAGRCAACGGATT-3′ and reverse, 3′-AAGCTTCCCGTTCTCAG-3′.

The PCR reaction condition included pre-denaturing at 94°C for 30 sec, then 35–40 cycle of 56°C for 30 sec, 72°C for 1 min. PCR products were then electrophoresed on a 1.5% agarose gel and visualized using a gel documentation system.

### Measurement of IL-6 and IL-8

The cells in triplicate were treated with the indicated doses of Tri-DAP and MDP or combination with TLR agonist or IFN-γ for 24 h and the culture supernatant was collected. The concentration of IL-6 and IL-8 in the culture supernatants were determined using a commercial ELISA kit (R&D Systems, Minneapolis, MN, USA)

### Western blotting

The cells (1×10^6^/well) were plated in 35-mm culture dishes. The cells were treated with 10 μg/ml of Tri-DAP and MDP and were lysed in buffer containing 1% Nonidet-P40 supplemented with a complete protease inhibitor cocktail (Roche) and 2 mM dithiothreitol. Lysates were resolved by 10% SDS-PAGE, transferred to a polyvinylidene fluoride (PVDF) membrane, and immunoblotted with primary antibodies against regular- and phospho-IκB-α, p38, ERK and JNK (Cell Signaling Technology, Inc., Beverly, MA, USA). After immunoblotting with secondary antibodies, proteins were detected with an enhanced chemiluminescence (ECL) reagent (Intron Biotechnology).

### Statistical analysis

The differences among the mean values of the different groups were assessed, and the values are expressed as the mean ± SD. All of the statistical calculations were performed by one-way ANOVA using the GraphPad Prism version 5.01 (GraphPad Software, San Diego, CA, USA). Values of P<0.05 were considered significant.

## Results

### Expression of NOD1 and NOD2 in PDL cells

**Figure 1:**
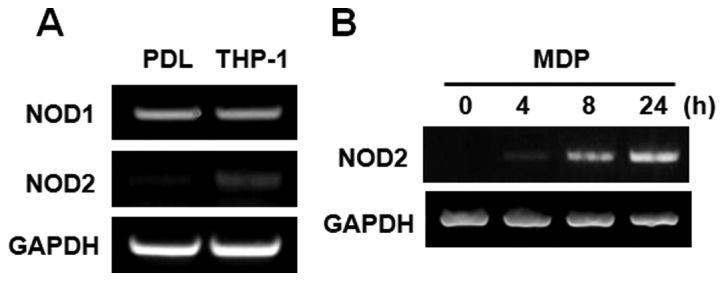
The gene expression levels of NOD1 and NOD2 in PDL cells. (A) The mRNA expression levels of NOD1, NOD2 and GAPDH were examined in human PDL cells by RT-PCR. THP-1 cells were used as a positive control. (B) PDL cells were stimulated with 10 μg/ml of MDP and total-RNA was extracted at the indicated times after stimulation. NOD2 expression levels were determined by RT-PCR.

The gene expression of NOD1 and NOD2 in PDL cells were examined by RT-PCR. THP-1 cells, human monocyte leukemia cells, were used as a positive control. The gene of NOD1 was strongly expressed in PDL cells, and NOD1 levels were comparable to that in THP-1 cells. In contrast, NOD2 expression was found to be low level in PDL cells, as compared to THP-1 cells (Fig. 1A). However, the stimulation with MDP, a specific NOD2 agonist, augmented the gene expression of NOD2 in a time-dependent manner in PDL cells (Fig. 1B).

### NOD1 and NOD2 stimulation leads to increased production of IL-6 and IL-8 in PDL cells

**Figure 2:**
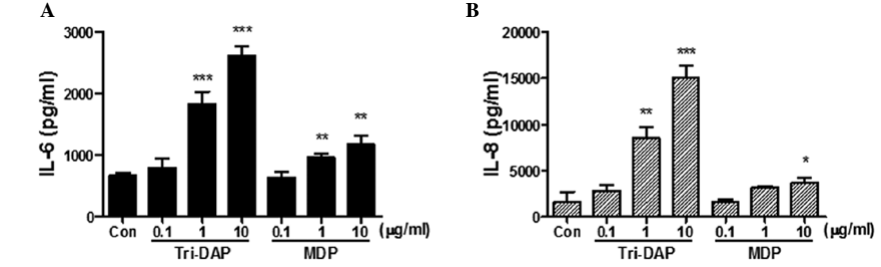
The production of IL-6 and IL-8 by PDL cells in response to Tri-DAP and MDP. The cells in triplicate were stimulated with the indicated doses of Tri-DAP or MDP for 24 h, and the levels of (A) IL-6 and (B) IL-8 in the culture supernatant were determined using a commercial ELISA kit. ^*^P<0.05; ^**^P<0.01; ^***^P<0.001.

To determine whether the stimulation of NOD1 and NOD2 leads to the production of pro-inflammatory cytokines/chemokines, the cells were treated with Tri-DAP (NOD1 agonist) and MDP (NOD2 agonist) and the production of IL-6 and IL-8 from culture supernatants was determined by ELISA. Both Tri-DAP and MDP can lead to increased production of IL-6 and IL-8 production in PDL cells in a dose-dependent manner (Fig. 2). Both IL-6 and IL-8 production was more increased by Tri-DAP than MDP, suggesting that NOD1 may play a more important role in the immune response of PDL cells than NOD2.

### Tri-DAP and MDP induce NF-κB and MAPK activation in PDL cells

**Figure 3:**
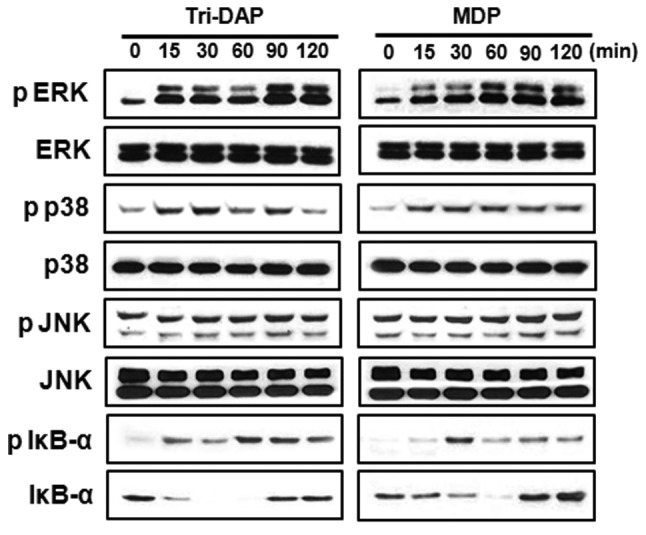
NF-κB and MAPK activation in PDL cells in response to Tri-DAP and MDP. The cells were stimulated with 10 μg/ml of Tri-DAP or MDP, and protein was extracted at the indicated time points. The total and phosphorylated forms of IκB-α, p38, JNK and ERK were examined by western blotting.

Sensing of microbial molecules by NOD1 and NOD2 induce the activation of NF-κB and MAPK in various cell types including macrophages (19–21). To determine whether NOD1 and NOD2 stimulation leads to NF-κB and MAPK activation in PDL cells, the cells were treated with Tri-DAP or MDP and extracts were prepared at different times after stimulation. Subsequently, immunoblotting was performed using antibodies that recognize activated forms of IκB-α, p38, JNK and ERK. Results showed that both Tri-DAP and MDP induced phosphorylation of IκB-α, p38 and ERK, but not JNK (Fig. 3). The kinetics of IκB-α phosphorylation and degradation were different between Tri-DAP and MDP. Fifteen minutes after stimulation, Tri-DAP induced phosphorylation of IκB-α and maximal phosphorylation was detected at 60 and 90 min after stimulation. However, MDP induced optimal phosphorylation of I-κBα at 30 min after stimulation, which was reduced after that time (Fig. 3).

### TLR stimulation enhances the gene expression of NOD1 and NOD2 and augments the production of IL-6 and IL-8 increased by Tri-DAP and MDP in PDL cells

**Figure 4:**
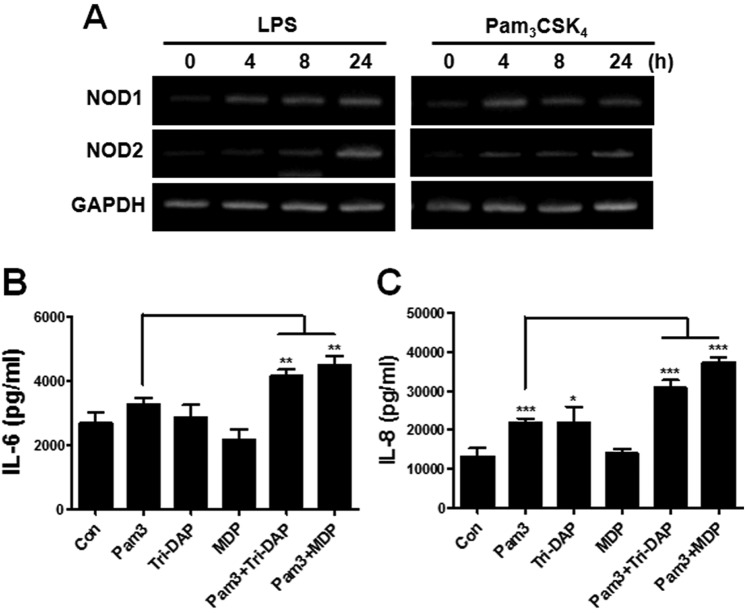
Upregulation of the gene expression of NOD1 and NOD2 by TLR agonists and synergism of NLR and TLR agonists for IL-6 production in PDL cells. PDL cells were stimulated with 1 μg/ml of LPS and Pam_3_CSK_4_ and total-RNA was extracted at the indicated times after stimulation. (A) The gene expression of NOD1 and NOD2 was determined by RT-PCR. PDL cells in triplicate were treated with indicated combination of Pam_3_CSK_4_ (0.1 μg/ml) and Tri-DAP or MDP (0.1 μg/ml) for 24 h and the levels of (B) IL-6 and (C) IL-8 in the culture supernatant were determined. ^*^P<0.05; ^**^P <0.01; ^***^P<0.001.

It has been known that TLRs synergize with NOD1 and NOD2 to produce cytokines in macrophages and dendritic cells (20,22). We first examined whether stimulation by TLRs affects the gene expression of NOD1 and NOD2 in PDL cells. The treatment of LPS (a TLR4 agonist) and Pam_3_CSK_4_ (a TLR2 agonist) could enhance the gene expression of NOD1 and NOD2 beginning at 4 h after stimulation (Fig. 4A). We next investigated whether activation of TLRs can augment the ability of PDL cells to produce IL-6 and IL-8 by Tri-DAP and MDP. Dose response experiments were performed to determine an appropriate dose of Pam_3_CSK_4_ to induce marginal production of IL-6 and IL-8. Results revealed that 0.1 μg/ml of Pam_3_CSK_4_ led to a minor increase of IL-6 and IL-8 production in PDL cells (data not shown), and this concentration was used for further experiments. For the synergism experiment, the cells were treated with indicated agonists alone or their combination for 24 h and IL-6 and IL-8 production was measured from culture supernatants. As shown in Fig. 4, combination treatment of Pam_3_CSK_4_ and Tri-DAP or MDP significantly augmented IL-6 or IL-8 production in PDL cells, as compared to the single agonist-treated group (Fig. 4B and C).

### IFN-γ synergizes with Tri-DAP and MDP to produce IL-6 in PDL cells

**Figure 5:**
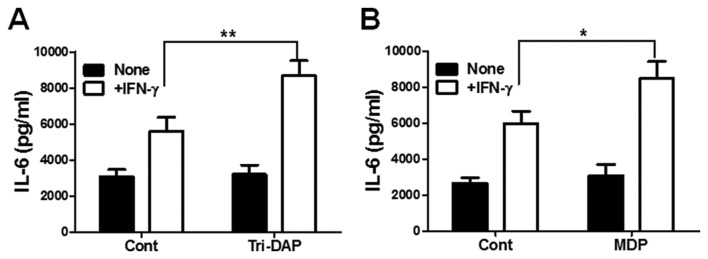
Tri-DAP and MDP augments IFN-γ-induced IL-6 production in PDL cells. PDL cells in triplicate were treated with IFN-γ (0.1 μg/ml), (A) Tri-DAP (0.1 μg/ml) and (B) MDP (0.1 μg/ml) alone or their combination for 24 h and the levels of IL-6 in the culture supernatant were determined. ^*^P<0.05; ^**^P<0.01.

Finally, we examined whether IFN-γ can augment Tri-DAP and MDP-induced cytokine production by PDL cells. IFN-γ alone could increase IL-6 production in PDL cells (Fig. 5A). In addition, co-stimulaion with Tri-DAP upregulated IL-6 production in PDL cells, as compared to IFN-γ or Tri-DAP alone (Fig. 5A). Furthermore, combination treatment with MDP and IFN-γ also enhanced IL-6 production by PDL cells, although a low dose of MDP alone (0.1 μg/ml) could not increase IL-6 production (Fig. 5B).

## Discussion

Recent studies have demonstrated that NOD1 and NOD2 are expressed in various cell types that exist within the oral tissues and play a role in triggering immune responses (23–25). In healthy gingival tissues, NOD1 and NOD2 exhibit stronger expression than TLRs (24). NOD1 and NOD2 are also expressed in various oral epithelial cells and the stimulation with iE-DAP and MDP upregulates the gene expression of β-defensin 2 (24). In human gingival fibroblasts, both NOD1 and NOD2 were strongly expressed and their agonists (FK156 for NOD1, MDP for NOD2) could increase the production of IL-6, IL-8 and MCP-1 via an NF-κB-dependent pathway (25). In addition, Hirao *et al* (23) showed the gene and protein expression of NOD1 and NOD2 in pulp fibroblasts and that iE-DAP and MDP could produce IL-8, suggesting that NOD1 and NOD2 are functionally expressed in pulp fibroblasts. Tang *et al* (26) showed the gene and protein expression and localization of NOD1 and NOD2 in human PDL fibroblasts. The activation of NOD1 and NOD2 led to the upregulation of tumor necrosis factor receptor-associated factor 6 (TRAF6) and pro-inflammatory cytokines in human PDL cells (26).

In the present study, we revealed that NOD1 is strongly expressed in PDL cells and the expression level is comparable to that in THP-1 cells. NOD2 expression was relatively weak in PDL cells. The expression level of NOD2 varies between cell types. The mRNA and protein of NOD2 was markedly expressed in hepatocytes, oral epithelial cells, and renal tubular epithelial cells (27–29), but was not expressed or was weakly expressed in intestinal epithelial cells (30). In addition, NOD2 expression seems to be regulated by specific treatment. Bacterial flagellin (a TLR5 agonist), *E. coli*, and IL-1β increased the gene expression of NOD2 in intestinal epithelial cells (30). Furthermore, in the presence of histamine, MALP-2 (a TLR2 agonist), peptidoglycan, and β-glucan also enhanced NOD2 gene expression in keratinocytes (31). In this study, the gene expression of NOD2 was upregulated by MDP stimulation, suggesting that NOD2 may be inducible in PDL cells. In addition, the activation of NOD1 and NOD2 with Tri-DAP and MDP led to IL-6 and IL-8 production and the activation of NF-κB and MAPK in PDL cells, indicating that NOD1 and NOD2 may be functionally expressed in PDL cells.

Previous studies showed that NOD1 and NOD2 have the synergistic or additive effect with TLRs to produce cytokines and chemokines in immune cells and mesothelial cells (20,21,32). These phenomena were also found in several epithelial cells. In oral epithelial cells, NOD1 and NOD2 agonists in combination with TLR agonists synergistically enhanced β-defensin 2 secretion (33). Moreover, LPS pretreatment enhanced the activation of NF-κB, ERK and JNK by MDP in hepatocytes (27). Likewise, in this study, NOD1 and NOD2 agonists (Tri-DAP and MDP) synergized with a TLR2 agonist Pam_3_CSK_4_ to produce IL-6 and IL-8 in PDL cells. Our results indicate that NOD1 and NOD2 may cooperate with TLRs to elicit immune responses in PDL cells.

IFN-γ is known to increase the expression of NOD1 and NOD2 in macrophages (34,35). In addition, IFN-γ is essential for a NOD1 agonist, KF1B-induced nitric oxide production in mesothelial cells (21). Therefore, we examined whether IFN-γ augments IL-6 production by NOD1 and NOD2 activation in PDL cells. Results showed that the co-stimulation with IFN-γ and Tri-DAP or MDP upregulated IL-6 production in PDL cells, as compared to IFN-γ or the agonist alone-treated group. These findings suggest that IFN-γ may enhance the innate immune response mediated by NOD1 and NOD2 signaling in PDL cells.

In conclusion, we reported here that NOD1 and NOD2 are functionally expressed in human PDL cells and the activation of these receptors can induce innate immune responses such as cytokine production and the activation of NF-κB and MAPK. In addition, our results revealed that TLRs can synergize with NOD1 and NOD2 to produce proinflammatory cytokines/chemokines. Similarly to immune responses, NOD1 and NOD2 signaling can mediate cellular physiological functions, such as proliferation and differentiation (36–38). Future studies should clarify the function of NOD1 and NOD2 on the cellular physiology of PDL cells.

## References

[1] Akira S Uematsu S Takeuchi O Pathogen recognition and innate immunity Cell 124 783 801 2006 16497588 10.1016/j.cell.2006.02.015

[2] Hoffmann JA The immune response of Drosophila Nature 426 33 38 2003 14603309 10.1038/nature02021

[3] Beutler B Eidenschenk C Crozat K Imler JL Takeuchi O Hoffmann JA Akira S Genetic analysis of resistance to viral infection Nat Rev Immunol 7 753 766 2007 17893693 10.1038/nri2174

[4] Medzhitov R Recognition of microorganisms and activation of the immune response Nature 449 819 826 2007 17943118 10.1038/nature06246

[5] Yu L Wang L Chen S Endogenous toll-like receptor ligands and their biological significance J Cell Mol Med 14 2592 2603 2010 20629986 10.1111/j.1582-4934.2010.01127.x PMC4373479

[6] Chen G Shaw MH Kim YG Nunez G NOD-like receptors: role in innate immunity and inflammatory disease Annu Rev Pathol 4 365 398 2009 18928408 10.1146/annurev.pathol.4.110807.092239

[7] Franchi L Warner N Viani K Nunez G Function of Nod-like receptors in microbial recognition and host defense Immunol Rev 227 106 128 2009 19120480 10.1111/j.1600-065X.2008.00734.x PMC2679989

[8] Inohara N Chamaillard M McDonald C Nunez G NOD-LRR proteins: role in host-microbial interactions and inflammatory disease Annu Rev Biochem 74 355 383 2005 15952891 10.1146/annurev.biochem.74.082803.133347

[9] Inohara N Koseki T Lin J del Peso L Lucas PC Chen FF Ogura Y Núñez G An induced proximity model for NF-κB activation in the Nod1/RICK and RIP signaling pathways J Biol Chem 275 27823 27831 2000 10880512 10.1074/jbc.M003415200

[10] Girardin SE Tournebize R Mavris M Page AL Li X Stark GR Bertin J DiStefano PS Yaniv M Sansonetti PJ Philpott DJ CARD4/Nod1 mediates NF-κB and JNK activation by invasive *Shigella flexneri* EMBO Rep 2 736 742 2001 11463746 10.1093/embo-reports/kve155 PMC1083992

[11] Hayden MS Ghosh S Signaling to NF-κB Genes Dev 18 2195 2224 2004 15371334 10.1101/gad.1228704

[12] Nishihara T Koseki T Microbial etiology of periodontitis Periodontol 2000 36 14 26 2004 15330940 10.1111/j.1600-0757.2004.03671.x

[13] Slots J Reynolds HS Genco RJ *Actinobacillus actinomycetemcomitans* in human periodontal disease: a cross-sectional microbiological investigation Infect Immun 29 1013 1020 1980 6968718 10.1128/iai.29.3.1013-1020.1980 PMC551232

[14] Socransky S Haffajee A Cugini M Smith C Kent R Jr Microbial complexes in subgingival plaque J Clin Periodontol 25 134 144 1998 9495612 10.1111/j.1600-051x.1998.tb02419.x

[15] Yamaji Y Kubota T Sasaguri K Sato S Suzuki Y Kumada H Umemoto T Inflammatory cytokine gene expression in human periodontal ligament fibroblasts stimulated with bacterial lipopolysaccharides Infect Immun 63 3576 3581 1995 7642293 10.1128/iai.63.9.3576-3581.1995 PMC173496

[16] Sun Y Shu R Li CL Zhang MZ Gram-negative periodontal bacteria induce the activation of Toll-like receptors 2 and 4, and cytokine production in human periodontal ligament cells J Periodontol 81 1488 1496 2010 20528699 10.1902/jop.2010.100004

[17] Sun Y Shu R Zhang MZ Wu AP Toll-like receptor 4 signaling plays a role in triggering periodontal infection FEMS Immunol Med Microbiol 52 362 369 2008 18328075 10.1111/j.1574-695X.2008.00386.x

[18] Fujii S Maeda H Wada N Kano Y Akamine A Establishing and characterizing human periodontal ligament fibroblasts immortalized by SV40T-antigen and hTERT gene transfer Cell Tissue Res 324 117 125 2006 16408200 10.1007/s00441-005-0101-4

[19] Kobayashi KS Chamaillard M Ogura Y Henegariu O Inohara N Nuñez G Flavell RA Nod2-dependent regulation of innate and adaptive immunity in the intestinal tract Science 307 731 734 2005 15692051 10.1126/science.1104911

[20] Park JH Kim YG McDonald C Kanneganti TD Hasegawa M Body-Malapel M Inohara N Núñez G RICK/RIP2 mediates innate immune responses induced through Nod1 and Nod2 but not TLRs J Immunol 178 2380 2386 2007 17277144 10.4049/jimmunol.178.4.2380

[21] Park JH Kim YG Shaw M Kanneganti TD Fujimoto Y Fukase K Inohara N Núñez G Nod1/RICK and TLR signaling regulate chemokine and antimicrobial innate immune responses in mesothelial cells J Immunol 179 514 521 2007 17579072 10.4049/jimmunol.179.1.514

[22] Tada H Aiba S Shibata KI Ohteki T Takada H Synergistic effect of Nod1 and Nod2 agonists with toll-like receptor agonists on human dendritic cells to generate interleukin-12 and T helper type 1 cells Infect Immun 73 7967 7976 2005 16299289 10.1128/IAI.73.12.7967-7976.2005 PMC1307098

[23] Hirao K Yumoto H Takahashi K Mukai K Nakanishi T Matsuo T Roles of TLR2, TLR4, NOD2, and NOD1 in pulp fibroblasts J Dent Res 88 762 767 2009 19734466 10.1177/0022034509341779

[24] Sugawara Y Uehara A Fujimoto Y Kusumoto S Fukase K Shibata K Sugawara S Sasano T Takada H Toll-like receptors, NOD1, and NOD2 in oral epithelial cells J Dent Res 85 524 529 2006 16723649 10.1177/154405910608500609

[25] Uehara A Takada H Functional TLRs and NODs in human gingival fibroblasts J Dent Res 86 249 254 2007 17314257 10.1177/154405910708600310

[26] Tang L Zhou XD Wang Q Wang Y Li XY Huang DM Expression of TRAF6 and pro-inflammatory cytokines through activation of TLR2, TLR4, NOD1, and NOD2 in human periodontal ligament fibroblasts Arch Oral Biol 56 1064 1072 2011 21457942 10.1016/j.archoralbio.2011.02.020

[27] Scott MJ Chen C Sun Q Billiar TR Hepatocytes express functional NOD1 and NOD2 receptors: a role for NOD1 in hepatocyte CC and CXC chemokine production J Hepatol 53 693 701 2010 20615568 10.1016/j.jhep.2010.04.026 PMC2930053

[28] Shigeoka AA Kambo A Mathison JC King AJ Hall WF da Silva Correia J Ulevitch RJ McKay DB Nod1 and nod2 are expressed in human and murine renal tubular epithelial cells and participate in renal ischemia reperfusion injury J Immunol 184 2297 2304 2010 20124104 10.4049/jimmunol.0903065 PMC3020136

[29] Sugawara Y Uehara A Fujimoto Y Kusumoto S Fukase K Shibata K Sugawara S Sasano T Takada H Toll-like receptors, NOD1, and NOD2 in oral epithelial cells J Dent Res 85 524 529 2006 16723649 10.1177/154405910608500609

[30] Begue B Dumant C Bambou JC Beaulieu JF Chamaillard M Hugot JP Goulet O Schmitz J Philpott DJ Cerf-Bensussan N Microbial induction of CARD15 expression in intestinal epithelial cells via toll-like receptor 5 triggers an antibacterial response loop J Cell Physiol 209 241 252 2006 16897777 10.1002/jcp.20739

[31] Kobayashi M Yoshiki R Sakabe J Kabashima K Nakamura M Tokura Y Expression of Toll-like receptor 2, NOD2 and dectin-1 and stimulatory effects of their ligands and histamine in normal human keratinocytes Br J Dermatol 160 297 304 2009 19016710 10.1111/j.1365-2133.2008.08897.x

[32] van Heel DA Ghosh S Butler M Hunt K Foxwell BM Mengin-Lecreulx D Playford RJ Synergistic enhancement of Toll-like receptor responses by NOD1 activation Eur J Immunol 35 2471 2476 2005 16021603 10.1002/eji.200526296

[33] Uehara A Takada H Synergism between TLRs and NOD1/2 in oral epithelial cells J Dent Res 87 682 686 2008 18573991 10.1177/154405910808700709

[34] Totemeyer S Sheppard M Lloyd A Roper D Dowson C Underhill D Murray P Maskell D Bryant C IFN-γ enhances production of nitric oxide from macrophages via a mechanism that depends on nucleotide oligomerization domain-2 J Immunol 176 4804 4810 2006 16585574 10.4049/jimmunol.176.8.4804

[35] Hisamatsu T Suzuki M Podolsky DK Interferon-γ augments CARD4/NOD1 gene and protein expression through interferon regulatory factor-1 in intestinal epithelial cells J Biol Chem 278 32962 32968 2003 12813035 10.1074/jbc.M304355200

[36] Kim HS Shin TH Yang SR Seo MS Kim DJ Kang SK Park JH Kang KS Implication of NOD1 and NOD2 for the differentiation of multipotent mesenchymal stem cells derived from human umbilical cord blood PLoS One 5 e15369 2010 21042538 10.1371/journal.pone.0015369 PMC2962653

[37] Petterson T Jendholm J Mansson A Bjartell A Riesbeck K Cardell LO Effects of NOD-like receptors in human B lymphocytes and crosstalk between NOD1/NOD2 and Toll-like receptors J Leukoc Biol 89 177 187 2011 20844241 10.1189/jlb.0210061

[38] Cruickshank SM Wakenshaw L Cardone J Howdle PD Murray PJ Carding SR Evidence for the involvement of NOD2 in regulating colonic epithelial cell growth and survival World J Gastroenterol 14 5834 5841 2008 18855982 10.3748/wjg.14.5834 PMC2751893

